# Association between Dietary Fibre Intake and Colorectal Adenoma: A Systematic Review and Meta-Analysis

**DOI:** 10.3390/ijerph18084168

**Published:** 2021-04-15

**Authors:** Daniele Nucci, Cristina Fatigoni, Tania Salvatori, Mariateresa Nardi, Stefano Realdon, Vincenza Gianfredi

**Affiliations:** 1 Nutritional Support Unit, Veneto Institute of Oncology IOV-IRCCS, Via Gattamelata 64, 35128 Padua, Italy; daniele.nucci@iov.veneto.it (D.N.); mariateresa.nardi@iov.veneto.it (M.N.); 2 Department of Pharmaceutical Sciences, University of Perugia, Via del Giochetto, 06122 Perugia, Italy; cristina.fatigoni@unipg.it (C.F.); tania.salvatori90@gmail.com (T.S.); 3 Digestive Endoscopy Unit, Veneto Institute of Oncology IOV-IRCCS, Via Gattamelata 64, 35128 Padua, Italy; stefano.realdon@iov.veneto.it; 4 School of Medicine, University Vita-Salute San Raffaele, 20132 Milan, Italy; 5 CAPHRI Care and Public Health Research Institute, Maastricht University, 6211 Maastricht, The Netherlands

**Keywords:** diet, fibre, colorectal, adenoma, systematic review

## Abstract

PubMed/Medline, Excerpta Medica dataBASE (EMBASE) and Scopus were searched in January 2021 in order to retrieve evidence assessing the association between dietary fibre intake and the risk of colorectal adenoma in adults. Preferred Reporting Items for Systematic Reviews and Meta-Analyses (PRISMA) guidelines were used for the reporting of results. Only primary observational studies were included. Publication bias was estimated through the Egger’s test and the visual inspection of the funnel plot. Heterogeneity between studies was calculated with I^2^ statistics. The search strategy identified 683 papers, 21 of which were included in our meta-analysis. Having evaluated a total of 157,725 subjects, the results suggest a protective effect of dietary fibre intake against colorectal adenoma. Effect Size (ES) was [0.71 (95% CI = 0.68–0.75), *p* = 0.000)]. Moderate statistical heterogeneity (Chi^2^ = 61.68, df = 23, I^2^ = 62.71%, *p* = 0.000) was found. Findings show a statistically significant (*p* = 0.000) and robust association between a higher intake of dietary fibre and a lower risk of colorectal adenoma, considering both the prevalent and incident risk. Moreover, the meta-regression analysis showed a borderline significant negative linear correlation between the amount of dietary fibre intake and colorectal adenoma. Lastly, we performed a subgroup analysis by sex, showing a higher protective effect for men.

## 1. Introduction

Colorectal cancer is the third most common cancer among men (after lung and prostate cancer) and the second among women (after breast cancer) worldwide, with approximately 2 new million cases (among both men and women) in 2020 [1]. Colorectal cancer is one of the few cancers for which a population screening program is in place practically all over the world [2]. There are several important reasons why colorectal cancer is suitable for population screening, including cancer progression from a preneoplastic (and subclinical) lesion (adenoma), the long lag time before invasive and malignant transformation, an easily detectable and treatable preneoplastic lesion, and the direct association between the stage of the disease and mortality [3]. It should be noted that colorectal adenoma is a proliferative dysplastic epithelial lesion that is harmful in most cases. It can have a malignant evolution based on the size, number, histology (grade of dysplasia) and duration in time [4]. Moreover, some other unmodifiable and modifiable factors might play an important role, such as age, ethnicity and genetics. Smoking, body mass index and diet seem to be the most important modifiable risk factors [5]. A high-fibre diet provide several plausible biological mechanisms that potentially provide a beneficial effect. Fibre might play a protective role through several mechanisms, including physical mechanisms, anti-inflammatory properties and prebiotic effects. Results from two extensive and recent meta-analyses confirm the protective role of fibre on colon [6] and rectal cancer risk [7]. However, despite the fact that adenoma as a preneoplastic lesion is recognized as a precursor of colorectal cancer, previous studies failed to univocally assess the role of dietary fibre intake and the risk of colorectal adenoma. These inconclusive results are probably due to a small population size, differences in the population’s characteristics, the adenoma site, the follow-up duration or the dose of fibre intake.

In this perspective, we performed the current systematic review with meta-analysis in order to collect and retrieve all relevant studies assessing the association between dietary fibre intake and the risk of colorectal adenoma. Moreover, we aimed to identify the amount of dietary fibre useful to prevent colorectal adenoma and also to estimate the risk among women and men and to evaluate the different risks of incident and prevalent colorectal adenoma.

## 2. Materials and Methods

The current systematic review with meta-analysis was accomplished following the methods recommended by the Cochrane Collaboration [8] and the Meta-analysis Of Observational Studies in Epidemiology (MOOSE) guidelines [9]. We followed the Preferred Reporting Items for Systematic Reviews and Meta-Analyses (PRISMA) [10] guidelines [11] to report the process and results. The review protocol was registered, in advance, on the International Prospective Register of Systematic Reviews (PROSPERO) (ID number: CRD42021230276), funded by the National Institute of Health Research (https://www.crd.york.ac.uk/prospero/, accessed on 8 January 2021). This systematic review was developed to answer the following research question: “Is dietary fibre intake associated with a lower (or higher) risk of colorectal adenoma?”

### 2.1. Search Strategy and Data Sources

Searches were independently carried out on the PubMed/Medline, Excerpta Medica dataBASE (EMBASE) and Scopus databases by two of the authors (VG and DN) in order to identify eligible studies. The structured computer literature search was built on a pre-determined combination of keywords, according to the type of database consulted. The search strategy was developed based on three domains: dietary fibre intake, colorectal adenoma and study design. Selected keywords were opportunely combined using Boolean operators. Moreover, we also built our search strategy using Medical Subject Headings (MeSH), text and title/abstract words. The strategy was first developed in PubMed/MEDLINE and then adapted for use in the other databases. The full search strategy is reported in Supplementary Table S1. The literature search on all the databases was carried out on the same day (10 January 2021) and included articles from inception (no time filter was used). Reference lists were also screened in order to identify additional relevant articles and experts in the field were consulted. The corresponding authors of included articles were contacted in case of missing data.

### 2.2. Inclusion/Exclusion Criteria

As recommended by the Cochrane Collaboration [12], a detailed description of inclusion/exclusion criteria, based on a Population, Exposure, Comparison, Outcomes and Study (PECOS) design [13], is reported in Supplementary Table S2. In brief, only those articles assessing the association between dietary fibre intake in healthy adults (both men and women) and the risk of colorectal adenoma were considered eligible. On the contrary, those studies that were conducted to assess the different outcomes or different fibre intake (for instance, supplementation) among subjects with comorbidities were excluded. With reference to the study design, only observational studies (cohort, case-control, cross-sectional or ecological studies) were considered for eligibility. Outcomes should be reported as risks [e.g., Odds Ratio (OR), Risk Ratio (RR) or Hazard Ratio (HR)], with their 95% Confidence Interval (CI 95%), or as a mean. Lastly, only articles published in English and with full-text available were considered.

### 2.3. Data Extraction

As had been done in previous studies [14,15], a two-step double-blind selection process was adopted by two researchers (DN and VG) to identify relevant articles. The selection process was firstly based on title and abstract screening; therefore the full-text was only obtained for potentially relevant studies. Data screening and data extraction was independently performed by two researchers (DN and CF) using a spreadsheet created in Microsoft Excel^®^ for Windows. The spreadsheet was pre-piloted on 5 randomly selected papers. This allowed us to increase methodological concordance. Selected articles and extracted data were compared, with any discrepancies being resolved throught discussion between the two researchers (DN and CF). If the disagreement persisted, a third researcher was consulted (VG). Both qualitative and quantitative data were extracted. Recorded quantitative data included sample size, study duration (expressed in years), amount of fibre intake and outcome (expressed as risk). Any adjusted estimated risks available in primary studies, were used preferentially. Qualitative data included: name of the first author, year of publication, country where the study was conducted, subjects’ characteristics, outcome measured, and tools used to assess dietary information and outcome diagnosis. Moreover, the presence of funding supporting the original research studies and information on conflict of interests (CoI) were also recorded.

### 2.4. Critical Appraisal

Two researchers (CF and DN) independently performed the critical appraisal, using the Newcastle-Ottawa Scale (NOS) [16] for observational studies. However, since the NOS did not provide a checklist for cross-sectional studies, we used a modified version [17] adapted to perform a quality assessment of cross-sectional studies. Using these criteria and based on a standard cut-off, we identified high-quality studies by means of the NOS if this was equal to or higher than 7 points.

### 2.5. Statistical Analysis

The effect size (ES) was calculated based on the odds ratio (OR), risk ratio (RR) and mean and sample size provided per study. It was estimated by OR and reported with its 95% CI. If papers presented a number of events (cases) among those exposed and not exposed and the mean value for dietary fibre intake for each group, the OR and CIs were computed from these data and included in the meta-analysis. The comparison was performed between subjects exposed to the highest intake of dietary fibre and subjects exposed to the lowest (or none) intake of dietary fibre consumption. Since the dietary fibre intake collected in each study was homogeneously reported, it allowed us to perform a meta-regression analysis. We applied a fixed and random model. A fixed model is used when the universe of studies is judged a priori to be sufficiently similar to those in the study sample, or when the number of included studies is small. The assumption behind the random effect model is that inferences are not limited to studies represented in the sample. In other words, it is presumed that the universe of studies contains studies with differences in their characteristics, and generalizations are based on studies that differ from those in the study sample. In this perspective, the random effect model is recommended if heterogeneity estimated values are considered high. Heterogeneity among included studies was evaluated through Chi^2^ and I^2^ tests. Heterogeneity was considered to be high when I^2^ values > 75%, moderate for I^2^ values ranging between 50% and 75%, low for values ranging between 25% and 50%, and no heterogeneity for values below 25%. The graphical evaluation of the Funnel plot and the Egger’s regression asymmetry test were used to estimate potential publication bias; statistical significance was set at *p* < 0.10 [18]. If any publication bias was detected, a trim and fill method, to search for missing studies to the right of overall, was used in order to adjust for publication bias [19]. The meta-analysis was performed using the Prometa3^®^ (Internovi, Cesena, Italy) software.

### 2.6. Sub-Group and Sensitivity Analysis

In order to exclude a potential overlapping effect due to the inclusion of studies referring to the same cohort of patients, a sensitivity analysis was run which only considered the study with the highest quality score (QS) or with the larger sample size in case of equal QS. We also performed the meta-analysis by excluding studies with computed OR. Furthermore, sensitivity analyses were conducted which only included studies with a follow-up (FU) equal to or higher than 9 years, with validated tools to assess dietary fibre intake, the type of diagnosis and a QS higher than 7. In addition, in order to corroborate the obtained results, sub-group analyses were performed based on the adenoma site, study design (case-control and cross-sectional vs. cohort studies) and sex.

### 2.7. Cumulative Analysis

A cumulative analysis is a sequential meta-analysis, intended to evaluate how adding one study at a time modifies the ES. We performed three cumulative analyses: the first one adding studies chronologically (starting from the first published analysis to the most recent publication), the second one based on the growing sample size (from the smallest to the biggest), and the third based on the ascending dose of dietary fibre intake (from the lowest to the highest). These types of analyses improve the potential consistency of results [20].

## 3. Results

### 3.1. Literature Search

**Figure 1:**
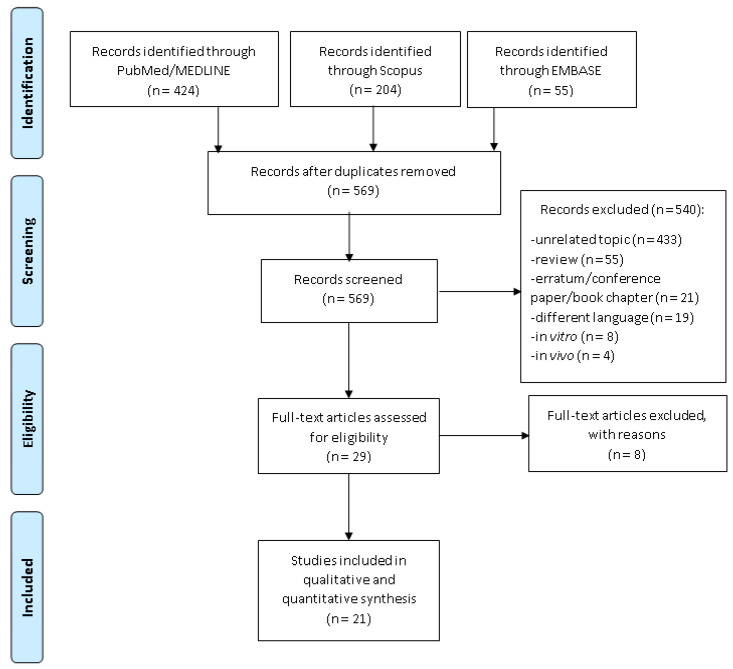
Flow diagram of the studies’ selection process.

We identified a total of 683 articles as follows: 424 in PubMed/Medline, 204 in the Scopus and 55 in EMBASE. After the removal of duplicates, a total of 569 original articles were preliminarily screened by title and abstract. From these, 549 were excluded because they were unrelated topics (*n* = 443), reviews (*n* = 55), not original studies (erratum, conference paper, commentary, letter and book chapters *n* = 21), in a different language (*n* = 19), in vitro studies (*n* = 8) and in vivo studies (*n* = 4). Overall, 29 studies were eligible, but 8 studies were excluded because of the reasons reported in Supplementary Table S2 [21,22,23,24,25,26,27,28]. Figure 1 depicts the flow diagram reporting the selection process. At the end of the screening process, 21 articles were included in the quantitative analysis [29,30,31,32,33,34,35,36,37,38,39,40,41,42,43,44,45,46,47,48,49]; however, because four papers reported separate data based on the adenoma site (colorectal, colon and rectal) [29,37,44,45], one paper reported the results for both incident and recurrent adenoma [37], another used a different control group (general population and hospital-based patients) [29], and two papers reported data stratified by sex [36,46], these were considered to be independent studies. Lastly, five studies [30,35,36,45,49] did not report the association between dietary fibre intake and colorectal adenoma as a risk, but as a number of events among those with higher and lower intake. Thus, ORs were computed for this reason. We considered them in the overall pooled estimate, but removed them in the sensitivity analysis.

### 3.2. Characteristics of Included Studies

**Figure 2:**
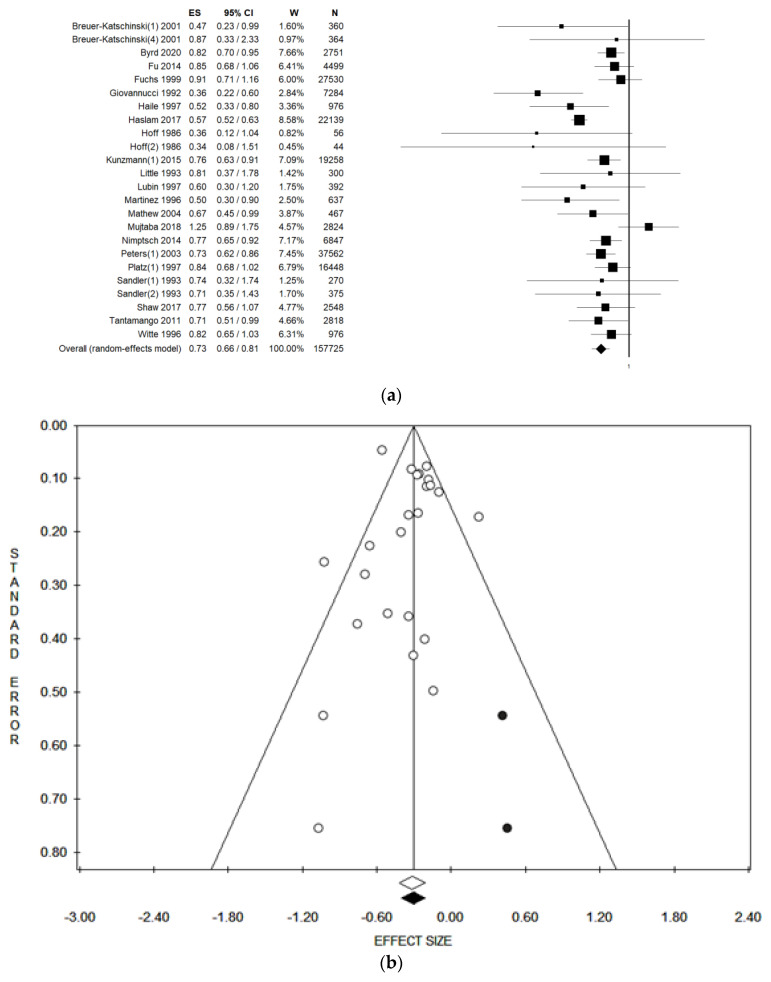
(**a**) Forest plot and (**b**) Funnel plot (after trim and fill method) of the meta-analysis comparing dietary fibre intake (lower vs higher intake) and risk of colorectal adenoma (random effect model). In (**a**) squares represent the effect size values of the individual studies. In (**b**) white dots represent single studies included. The black dots represent estimated studies after the trim and fill method. The white diamond represents the overall effect size of the included studies. The black diamond represents the estimated overall effect size after the trim and fill method.

Table 1 and Table 2 show the characteristics of the studies included in the meta-analysis in alphabetical order. Supplementary Table S3 shows the quality evaluation. The first included study was published in 1986 [36], whereas the most recent was published in 2020 [30], however, the period with the highest number of publications on the topic were the decades 1990–2000 and 2010–2020. Almost all the included studies were conducted in the United States of America (USA), but one was conducted in Israel [39] and two in Europe (Germany [29] and Norway [36], respectively). As regards the study design, 13 studies were case-control [29,30,31,34,36,38,39,40,41,42,44,46,49], seven were cohort studies [32,33,35,37,43,45,48], and one was a cross-sectional study [47]. Only considering cohort studies, the follow-up (FU) period ranged between 2 years to 26 years [43]. The sample size ranged between 100 [36] and 37,562 participants [44], with a population age ranging from 18 to 79 years. More than half of the included studies (*n* = 13) used a validated self- administered Food Frequency Questionnaire (FFQ) to assess dietary fibre intake [30,31,32,33,34,35,37,41,42,43,44,45,49], one study used a diet history questionnaire [47], and another used the food diary [36]. Furthermore, five studies performed an interview [29,38,39,40,46], however three studies did not provide information on validation [40,44,48]. When pooling data in meta-analysis, higher dietary fibre intake was associated with a lower risk of colorectal adenoma [in the fixed effect model, pooled ES = 0.71 (95% CI = 0.68–0.75), *p* = 0.000; in the random effect model, pooled ES = 0.73 (95% CI = 0.66–0.81), *p* = 0.000; based on 157,725 participants, with moderate statistical heterogeneity (Chi^2^ = 61.68, df = 23, I^2^ = 62.71%, *p* = 0.000)] (Figure 2a) and potential publication bias (Figure 2b), at which trim and fill method was applied (Table 3).

**Figure 3:**
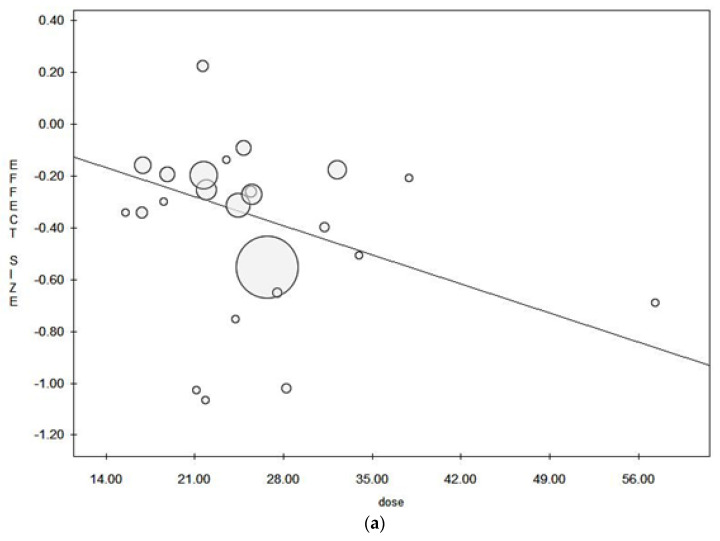

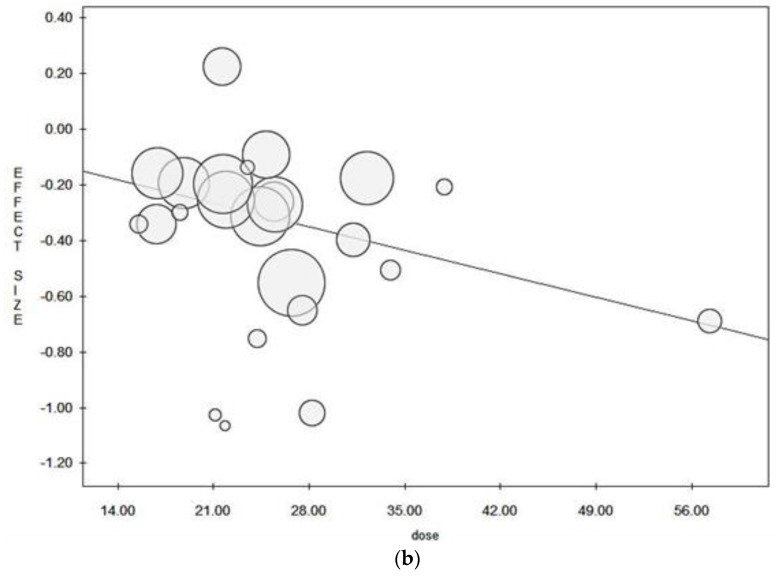
Meta-regression plot for (**a**) fixed effect model and (**b**) random effect model.

No publication bias was found when considering the fixed effect model, as demonstrated by the symmetry of the Funnel plot and confirmed by Egger’s linear regression Test (Intercept 0.12, t = 0.21, p = 0.838) (Figure 2b). However, a potential publication bias was found when the random effect model was considered. In this case, we applied the trim and fill method and, after trimming 2 studies, we obtained an estimated pooled ES = 0.71 (95% CI = 0.68–0.75), p = 0.000 in the fixed effect model and an estimated pooled ES = 0.74 (95% CI = 0.67–0.82), p = 0.000 in the random effect model (Table 3). With regard to the dose-response meta-regression analysis, all studies reported dietary fibre as grams per day (g/d), but two studies reported dietary fibre as g/1000 kcal/d [30,37]. In view of this, we assumed a standard daily energy intake of 2000 kcal and, for this reason we multiplied the reported intake by two. Figure 3 shows the meta-regression plot of the log OR (colorectal adenoma) on the dose of dietary fibre intake, using the fixed effect (Figure 3a) and the random effect models (Figure 3b). The size of the circles (identifying the included studies) denotes the study’s weight which is represented by the inverse of the within-study variance in the fixed model, and the total variance for each study in the random effect model. The line shows the predicted values where, in the fixed effect model, the intake of dietary fibre showed negative weak-border line significant correlation with colorectal adenoma (Y = −0.06, z = −0.02, p = 0.056), whilst in the random effect model, the linear correlation was not significant (Y = −0.01, z = −0.01, p = 0.095).

### 3.3. Sub-Group and Sensitivity Analysis

A series of sensitivity analyses were conducted in order to confirm the robustness of our results. Firstly, we removed those studies that used the same cohort in order to reduce ES overestimation due to the potential overlapping effect. In particular, four studies were excluded from the analysis [30,33,35,44], thus obtaining similar results compared to the main analysis but with no statistical heterogeneity (I^2^ = 13.00%, *p* = 0.295) (Table 3). Potential publication bias was found, as confirmed by Egger’s Linear Regression Test (Intercept −0.89, t = −1.93, *p* = 0.070). Also, in this case, a trim and fill method was applied by trimming six studies on the right and the results did not change [fixed effect estimated ES = 0.79 (95% CI = 0.74 − 0.85), *p* = 0.000; the random effect model estimated ES = 0.82 (95% CI = 0.77 − 0.91), *p* = 0.000)].

Secondly, we removed studies for which OR was computed based on data reported in the original studies [30,35,36,45,49], obtaining similar results with no heterogeneity and an absence of potential publication bias as confirmed by Egger’s Linear Regression Test (Table 3). Thirdly, only those studies with an FU equal to or higher than 9 years were included; the results based on 53,827 participants did not materially change and no statistical heterogeneity (I^2^ = 0.00%, *p* = 0.018) was found (Table 3). No publication bias was found as confirmed by Egger’s Linear Regression Test (Intercept 0.15, t = 0.07, *p* = 0.950). Fourthly, results were not affected by limiting the analysis to only include studies that used validated tools to assess dietary fibre intake; however, a high statistical heterogeneity was found (Table 3) but with no publication bias.

Stronger results were obtained when limiting the pooling of studies by only using colonoscopy and histopathological confirmation for the diagnosis of adenoma and for studies with a quality score higher than 7 [low statistical heterogeneity (I^2^ = 38.10%, *p* = 0.007) but with potential publication bias (Egger’s Linear Regression Test Intercept −0.99, t = −1.96, *p* = 0.065); however, the trim and fill method, by trimming 6 studies on the right, did not change results: in the fixed effect model, the pooled ES was 0.81 (95% CI = 0.76–0.86), *p* = 0.000; in the random effect model ES was 0.82 (95% CI = 0.73–0.91) *p* = 0.000]. In order to corroborate the results obtained, sub-group analyses were additionally performed, based on the adenoma site (colon and rectal separately), study design (case-control/cross-sectional and cohort studies) and sex. Results also remained similar in these cases. We detected a potential publication bias in the subgroup analyses by study design and sex when only including case control/cross-sectional studies and men, respectively. However, ES estimated after the trim and fill method did not change. Data are shown in Table 3. Results should be interpreted with caution for adenoma site and sex since a few studies for each analysis were pooled and also due to the low number of stratified analyses conducted in the original studies retrieved.

### 3.4. Cumulative Analysis

**Figure 4:**
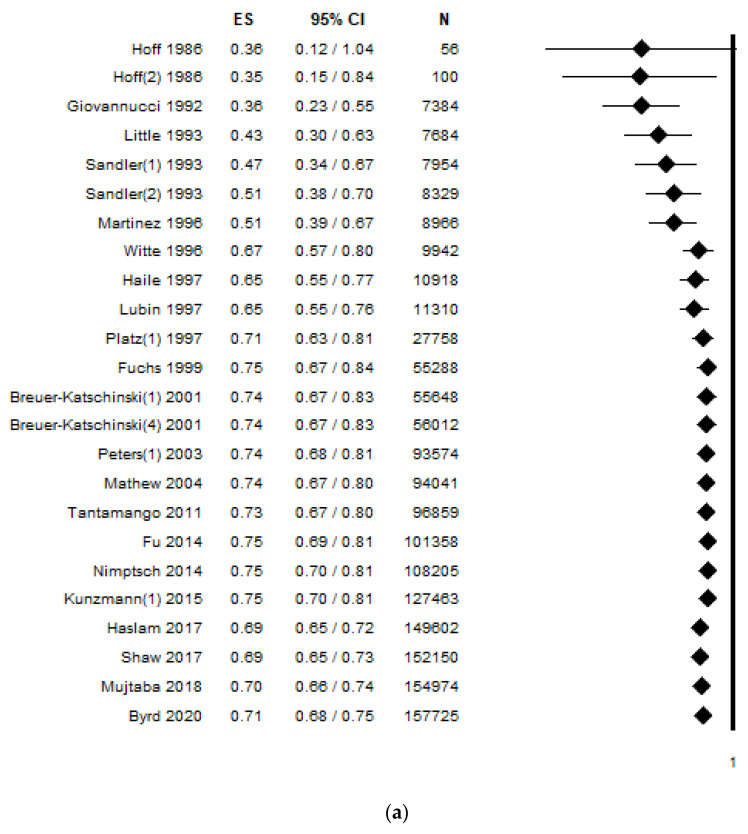

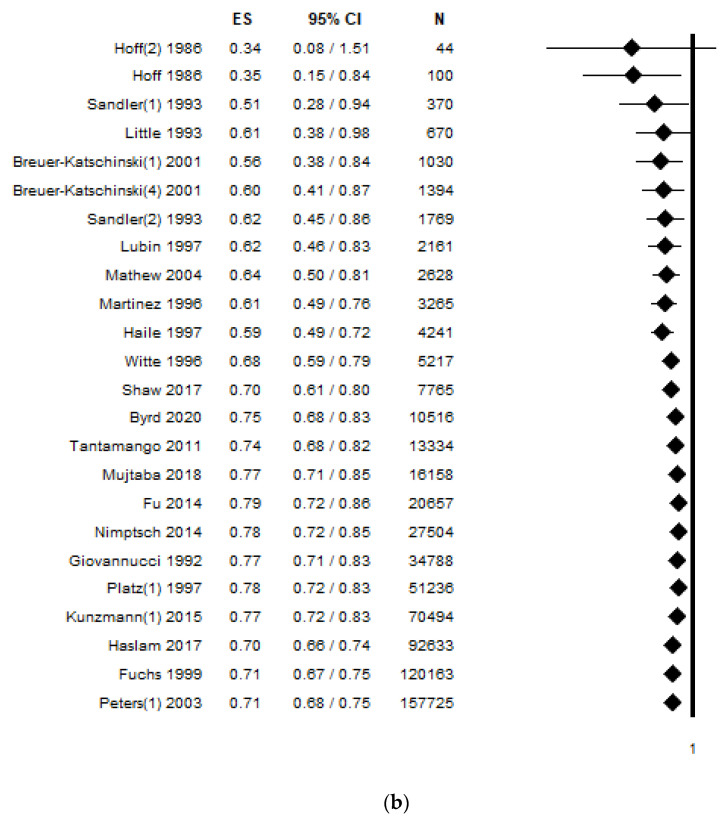

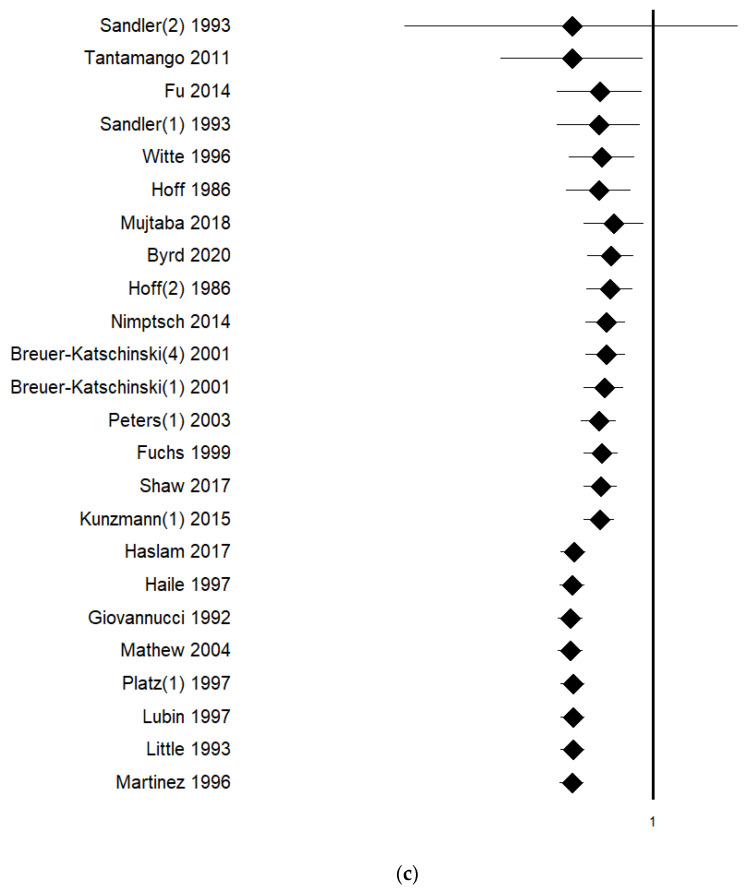
Forest plot of the cumulative analysis by (**a**) year of publication (from the first to the most recent published study), (**b**) by sample size (from the smallest to the largest), (**c**) dietary fibre dose (from the lowest to the highest intake) between dietary fibre intake and risk of colorectal adenoma. Diamonds represent the effect size estimated using the cumulative analysis calculated adding one study at a time.

The cumulative analysis in Figure 4a reveals that early studies, even if suggesting an inverse association between fibre intake and risk of colorectal adenoma, had a wide 95% CI which decreased in the late 1990s and relatively stabilized during the first decade of 2000. However, a really narrow 95% CI and a consistent ES was obtained during the second decade of 2000. With reference to the cumulative analysis by sample size (Figure 4b), studies with a sample size smaller than 1000 subjects, even if establishing an inverse statistically-significant association, had a wide 95% CI. As expected, a larger sample size contributed to stabilizing the results both by reducing the 95% CI and by corroborating the ES value. Considering the cumulative analysis by dietary fibre dose, ES was higher when studies examined a dietary dose lower than 26 g/d. Conversely, ES was lower and stable and with a narrow 95% CI, when higher dietary fibre intake was considered (Figure 4c).

## 4. Discussion

This is an extensive systematic review and meta-analysis of observational studies conducted by searching three different databases (PubMed/Medline, Scopus and EMBASE) and assessing the association between dietary fibre intake and the risk of colorectal adenoma. Our meta-analysis of 21 studies in total found an approximate 30% risk reduction of adenoma associated with a higher intake of dietary fibre. This result was confirmed in both the fixed and random effect models. Moreover, meta-regression analysis was performed since the original studies reported dietary fibre intake homogeneously. Meta-regression analysis predicts the changes of the outcome (colorectal adenoma) for a unit increase in dietary fibre intake. Our results showed a border-line significant negative linear correlation between the amount of dietary fibre intake and colorectal adenoma (the higher the intake of dietary fibre, the lower the risk of colorectal adenoma). This observation is in line with the cumulative analysis by dietary fibre dose, according to which the lowest risk of adenoma was associated with a dietary fibre intake equal to or higher than 26 g/d. However, it should be noted that the ES from the pooling of the 21 studies was associated with moderate heterogeneity and a potential publication bias. For these reasons, we performed several sensitivity analyses and the trim and fill method was applied. With regard to the potential publication bias, the trim and fill methods trimmed two possible studies on the right, however, the result did not change. Looking at the subgroup analysis by sex, a higher protective effect for men was found when compared to women, yet there was higher heterogeneity in men than in women. Furthermore, the highest strength of the association between dietary fibre intake and colorectal adenoma was found when focusing on cohort studies wherein higher heterogeneity was found when compared to case-control/cross-sectional studies. This is probably because a different duration of FU was considered among the original pooled cohort studies, and this hypothesis can be confirmed when considering a sensitivity analysis limited to 9 years (or more) of FU, where heterogeneity dramatically dropped. Moreover, the natural history of colorectal adenoma should be considered when interpreting these results. On the one hand, colorectal adenoma is characterized by a long latency period, which might not be better appraised in short cohort studies, nor in long cohort studies, where maintaining an FU might be difficult while increasing the risk of selection bias. On the other hand, case-control studies are more prone to potential recall bias. However, the high number of retrieved studies and consequently, the large sample size, might mitigate the risk. An important aspect that should be considered is that the vast majority of included studies were conducted in the USA, a population that largely did not meet healthy dietary guidelines, and where the eating pattern of approximately three-fourths of the population is low in vegetables, fruits, dairy, and oils and rich in refined grain, proteins, saturated fats, sodium and total calories [50]. This dietary pattern is frequently associated with several preventable, diet-related chronic diseases, including cancers. Based on this, we can speculate that the healthy beneficial effects of dietary fibre intake might be higher with respect to what we found in this meta-analysis if assessed in a population with a more Mediterranean—or, generally speaking, healthier—dietary pattern. It should be considered that the most relevant source of dietary fibre derives from vegetables, legumes and fruits which have been shown to prevent colorectal cancer [51] and which are heterogeneous in respect to their composition; therefore, it can be assumed that they have various anticarcinogenic properties. They are rich in bioactive compounds such as vitamins, anti-oxidants and polyphenols. Previous in vitro studies showed that some of these polyphenols, such as sulforaphane and epigallocatechin, are able to reprogram gene expression through epigenetic modification, thus reverting cancer progression [52,53]. Moreover, it should be considered that almost all the included studies used FFQs to assess dietary fibre intake, and even if they were frequently validated, it is difficult to precisely estimate the intake, often resulting in underestimation. Furthermore, our results suggest that there might be differences in the responses to fibre by sex.

### 4.1. Potential Biological Mechanisms

Since colorectal adenomas are considered to be potential precancerous lesions, they are likely to share a common etiopathogenesis with colorectal cancer.

According to the European Food Safety Authority (EFSA) dietary fibre is “non-digestible carbohydrates plus lignin, including non-starch polysaccharides, fructo-oligosaccharides, galactooligosaccharides, other resistant oligosaccharides and resistant” [54]. Major food sources for dietary fibre are cereals/grains, vegetables, fruits and legumes. Based on its components, previous studies suggested to differentiate dietary fibre into “soluble” and “insoluble”. Such distinction was used to differentiate between viscous, soluble types of fibre (e.g., pectins) and insoluble components such as cellulose. Even if the distinction was mainly proposed to identify different patterns of beneficial effects, it should be noticed that both soluble and insoluble components have different and synergic advantages. The insoluble fibre is mainly responsible for the increase in stool bulk, important for reducing transit time and diluting carcinogens in the lumen by means of both reducing exposure to carcinogens and lowering secondary bile acid production [5]. The soluble fibre, instead, seems to be implicated in the wellbeing of microbiota through fibre fermentation which, in turn, is able to promote the production of short-chain fatty acids (SCFA) and which, by lowering the colonic pH, might inhibit pathogenic microorganisms and increase the absorption of some nutrients [55]. In addition, experimental studies have shown that butyrate, SCFA, has anti-proliferative effects, promotes colon motility and induces apoptosis [56]. The consequent reduction in cholesterol, and insulin resistance, seem to inherently reduce the risk of colorectal cancer. Furthermore, dietary fibre intake seems to also promote the eubiosis of the gut microbiota ecosystem [57]. On the contrary, dysbiosis (increased number of harmful bacteria in the gut) seems to be associated with an increased release of enterotoxins that alter the immune system, inducing the production of pro-inflammatory cytokines responsible for the disease status [58], including colorectal cancer [59].

Considering the beneficial effects of both dietary fibre components, and based on the suggestion provided by Food and Agriculture Organization (FAO) and World Health Organization (WHO) stating that the above mentioned distinction should be overcome as solubility does not always predict physiological effects [60], we did not perform a separate analysis among soluble and insoluble fibre. Indeed, the potential role of fibre in preventing colorectal adenoma and then cancer could be attributed to all of the mentioned mechanisms, mainly on account of the dietary fibre heterogeneity in chemical composition, physicochemical properties, and solubility. On the other hand, selectively focusing on one of the two components and consequently on a particular food source might lead to a reduction in diet variety. In light of this, international healthy dietary guidelines consider dietary fibre as a single entity [61] and recommend satisfying the daily dietary fibre intake of at least 30 g, derived from a varied and balanced diet, rich in plant-based foods, such as wholegrains, legumes, non-starchy vegetables and fruit [62], as the Mediterranean diet advocates [63]. Nevertheless, in our society, the increased rate of colorectal adenoma (and cancer) can be attributed, among the others, to unhealthy lifestyles, including the so-called Western diet. This dietary pattern is characterized on one hand by a high intake of refined grains, sugars, salt, saturated and trans-fatty acids mainly due to a high consumption of ultra-processed food [64,65], and on the other hand by a low intake of dietary fibre. In this respect, previous studies showed that a low intake of fibre along with a high consumption of typical western diet food increase the risk of dysbiosis, which in turn can be responsible for a lower production in SCFA [66,67].

### 4.2. Strengths and Limitations

This meta-analysis is affected by some limitations directly stemming from the included studies. First of all, dietary fibre intake was self-reported, most of the time using FFQ which, even if validated, cannot prevent recall bias or social desirability bias, thus both resulting in mis-reporting. Secondly, information on cooking methods were not assessed and reported in the original studies, and recent evidence showed a modification in the fibre structure and biological effect based on the processing method used [68,69]. Thirdly, in the main analysis, the funnel plot confirmed by the Egger’s test showed a potential publication bias. However, when applying the trim and fill methods the estimated ES did not change when compared to the observed ES. Another possible limitation is the language used (only articles published in English), which can lead to a bias in the selection of studies. Since English is the commonly-used scientific language, we believe that this did not affect our results as the most relevant and high-quality articles are published in English. Despite the above-mentioned limitations, our review with meta-analysis has important strengths. Worth highlighting is that this is a systematic and extensive review of available evidence, conducted according to the main guidelines. Furthermore, we conducted several sensitivity and sub-group analyses that make our results stronger and consistent. All the sub-group analyses performed offer a deep understanding of the association, useful for public health experts, dietitians and clinicians—the former involved in issuing health-related policies and campaigns and the latter in educating and assisting patients. Moreover, we were able to perform a meta-regression analysis as well as a cumulative analysis, confirming the association between a higher intake of dietary fibre and a lower risk of colorectal adenoma. Lastly, in our meta-analysis we pooled ES with a higher level of adjustment than was reported in each original study.

## 5. Conclusions

To conclude, the results of our systematic review with meta-analysis show a statistically significant and robust association between a higher intake of dietary fibre and a lower risk of colorectal adenoma, both considering a prevalent and incident risk of adenoma (as confirmed by the subgroup analysis by study design). These results are extremely important because we were able to estimate the reduction in the risk of the development of a preneoplastic lesion as a result of a higher dietary fibre intake, which can be considered as a valid healthy dietary recommendation. Given the relatively simple implementation of this dietary behaviour, our findings confirm the importance of reinforcing this knowledge and awareness among both health care professionals (including, but not limited to those involved in nutritional educational programmes) and the general population.

## Figures and Tables

**Table 1:** Qualitative characteristics of included studies, reported in alphabetical order.

Author, Year [Ref]	Country	Study Period	Study Design	Population Characteristics	Tool	Diagnostic Assessment	Funds	Conflicts of Interest
Breuer-Katschinski, 2001 [29]	Germany	2 years	Case-controls	Patients from five major hospitals in Essen; controls were selected from among hospital patients and stratified by sex	Personal interview not validated	Endoscopy and histology	yes	n.a.
Breuer-Katschinski, 2001 (a) [29]	Germany	2 years	Case-controls	Patients from five major hospitals in Essen; controls were selected from among the general population and stratified by sex	Personal interview not validated	Endoscopy and histology	yes	n.a.
Byrd, 2020 [30]	USA	1991–19941994–19972002	Case-controls	MAP I and MAP Il	Validated self-administered 61-FFQs and 98-FFQ	Colonoscopy and histology	yes	yes
Fu, 2014 [31]	USA	7 years	Case-controls	TCPS	Validated self-administered 108-FFQ	Colonoscopy and histology	yes	no
Fuchs, 1999 [32]	USA	16 years	Cohort	Without history of cancer, IBD, or familial polyposis	Validated self-administered 136-FFQ	Medical records	n.a.	n.a.
Giovannucci, 1992 [33]	USA	2 years	Cohort	HPF	Validated self-administered 131-FFQ	Endoscopy and histology	n.a.	n.a.
Haile, 1997 [34]	USA	2 years	Case-controls	Screening sigmoidoscopy subjects from 2 Southern California Kaiser Permanente Medical Centers	Validated 126-item semi-quantitative FFQ	Sigmoidoscopy and histology	yes	n.a.
Haslam, 2017 [35]	USA	7 years	Cohort	PLCO	Validated questionnaire 137-FFQ	Sigmoidoscopy and histology	no	yes
Hoff, 1986 [36]	Norway		Case-controls	Endoscopic population screening study	Food diary for 5 consecutive days	Rectosigmoidoscopy	n.a.	n.a.
Kunzmann, 2015 [37]	USA	13 years	Cohort	PLCO outcome stratified by adenoma site (incident)	Validated self-administered 137-FFQ	Sigmoidoscopy and histology	n.a.	no
Kunzmann, 2015 (a) [37]	USA	13 years	Cohort	PLCO outcome stratified by adenoma site (recurrent)	Validated self-administered 137-FFQ	Sigmoidoscopy and histology	n.a.	no
Little, 1993 [38]	UK	7 years	Case-controls	Subjects recruited in a colorectal cancer screening trial in Nottingham	Interview conducted at the subject’s home by specially trained interviewers	Colonoscopy and histology	yes	n.a.
Lubin, 1997 [39]	Israel	3 years	Paired Case-controls	Subjects identified in the SPGD at the Tel Aviv Medical Center	180-item questionnaire (interview)	Endoscopy and histology	n.a.	n.a.
Martìnez, 1996 [40]	USA	2 years	Case-controls	Population without history of colorectal polyps and familial polyposis	138-FFQ (interview) validation n.a.	Sigmoidoscopy or colonoscopy and histology	n.a.	n.a.
Mathew, 2004 [41]	USA	2 years	Case-controls	Subjects with new or recurrent adenomas in a study conducted at the NNMC	Validated self-administered 100-item	Sigmoidoscopy or colonoscopy and histology	n.a.	n.a.
Mujtaba,2018 [42]	USA	1991–19941994–19972002	Case-controls	CPRU MAP I MAP Il	Validated self-administered 61-FFQs	Colonoscopy and histology	n.a.	no
Nimptsch, 2014 [43]	USA	9 years	Cohort	NHS II	Validated self-administered 131-FFQ	Medical record	yes	n.a.
Peters, 2003 [44]	USA	7 years	Case-controls	PLCO	Self-administered 137-FFQ (adaptation from previous validated FFQ)	Endoscopy and histology	n.a.	n.a.
Platz, 1997 [45]	USA	8years	Cohort	HPF	Validated self-administered 131-FFQ)	Sigmoidoscopy or colonoscopy and histology	yes	n.a.
Sandler, 1993 [46]	USA	2 years	Case-controls	Subjects who underwent colonoscopy at the University of North Carolina Hospitals	Validated quantitative food frequency questionnaire (interview)	Sigmoidoscopy or colonoscopy and histology	n.a.	n.a.
Shaw, 2017 [47]	USA	7 years	Cross-sectional	FMCCSC	DHQ I or II	Colonoscopy and histology	yes	no
Tantamango, 2011 [48]	USA	26 years	Cohort	AHS-1 and AHS-2	Self-administered 55-FFQ validation n.a.	Self-reported	yes	no
Witte, 1996 [49]	USA	2 years	Paired Case-controls	Subjects free of invasive cancer, IBD and familial polyposis	Validated self-administered 126-FFQ	Sigmoidoscopy and histology	n.a.	n.a.

n.a.: not available; no: declared, but conflicts of interest absent; yes: declared and present. AHS: Adventist Health Study; CPRU: Cancer Prevention Research Unit Study; DHQ: Diet History Questionnaire; FFQ: food frequency questionnaire; FMCCSC: Forzani &MacPhail Colon Cancer Screening Centre; IBD: Inflammatory bowel disease; HPF: Health Professionals Follow-up Study MAP: Markers of Adenomatous Polyps; NHS: Nurses’ Health Study; NNMC: National Navy Medical Center; NSAID: Nonsteroidal anti-inflammatory drug; PA: Physical Activity; PLCO: Prostate, Lung, Colorectal and Ovarian Cancer Screening Trial; SAF: Saturated fatty acids; SPGD: Screening Program of the Gastroenterology Department; TCP: Tennessee Colorectal Polyp Study; TEn: Total Energy intake; UK: United Kingdom; USA: United States of America.

**Table 2:** Quantitative characteristics of included studies, reported in alphabetical order.

Author, Year [Ref] (Number of Stratified Analysis)	Total Sample ^	Sex	Age (In Years) Mean ± SD	Dietary Fibre Intake Mean ± SD	Outcome	n. Subjects at the Highest Fibre Intake	Highest Dietary Fibre Intake	Effect Size (95% CI) *p*	Adjustment
Breuer-Katschinski, 2001 [29] (1)	Ca: 182 Co I: 178	Ca: M = 94 Co I: M = 88	Ca: 63.8 ± 9.9 Co I: 63.4 ± 9.8	Ca: 23.01 ± 7.68 g/d Co: 24.27 ± 8.09 g/d	colorectal adenoma	n.a.	n.a.	RR 0.47 (0.23–0.99) *p* < 0.05	TEn, BMI, and social class
Breuer-Katschinski, 2001 [29] (2)	Ca: 94 Co I: 88	Ca: M = 94 Co I: M = 88	Ca: 63.8 ± 9.9 Co I: 63.4 ± 9.8	n.a.				RR 0.16 (0.05–0.57) *p* < 0.05	
Breuer-Katschinski, 2001 [29] (3)	Ca: 88 Co I: 90	Ca: F = 88 Co I: F = 90	Ca: 63.8 ± 9.9 Co I: 63.4 ± 9.8	n.a.				RR 0.66 (0.23–1.86) *p* = n.s.	
Breuer-Katschinski, 2001 (a) [29] (4)	Ca: 182 Co II: 182	Ca: M = 94 Co II: M = 92	Ca: 63.8 ± 9.9 Co II: 64.2 ± 9.9	Ca: 23.01 ± 7.68 g/d Co: 23.60 ± 6.75 g/d	colorectal adenoma	n.a.	n.a.	RR 0.87 (0.33–2.33) *p* = n.s.	TEn, BMI, and social class
Breuer-Katschinski, 2001 (a) [29] (5)	Ca:94 Co II: 92	Ca: M = 94 Co II: M = 92	Ca: 63.8 ± 9.9 Co II: 64.2 ± 9.9	n.a.				RR 0.38 (0.14–1.05) *p* = n.s.	
Breuer-Katschinski, 2001 (a) [29] (6)	Ca: 88 Co II: 90	Ca: F = 88 Co II: F = 90	Ca: 63.8 ± 9.9 Co II: 64.2 ± 9.9	n.a.				RR 0.86 (0.29–2.29) *p* = n.s.	
Byrd, 2020 [30]	Ca: 765 Co: 1986	Ca: M = 462 Co: M = 846	Ca: 58.2 ± 9.2 Co: 54.5 ± 10.9	Ca: 10.9 ± 3.7 g/1000 kcal/d Co: 11.3 ± 3.9 g/1000 kcal/d	colorectal adenoma	n.a.	n.a.	OR 0.82 (0.70–0.95) *p* = 0.01	none
Fu, 2014 [31]	Ca: 1315 Co: 3184	Ca: M = 913 Co: M = 1732	Ca: 59.2 Co: 57.5	Ca: 17.0 ± 1.0 g/d Co: 17.3 ± 1.0 g/d	colorectal adenoma	Ca: 278	24.8 g/d	OR 0.85 (0.68–1.06) *p* = 0.06	Age, sex, sites, education, smoking, PA, food supplement, dietary calcium and folate intake, and TEn
Fuchs, 1999 [32]	27,530 Ca: 787	only F	49	n.a.	colorectal adenoma	Ca: 212	24.9 ± 5.5 g/d	RR: 0.91 (0.71–1.16) *p* = 0.36	Age, smoking, BMI, PA, aspirin use, family history of CRC, history of colorectal adenoma, red meat intake, alcohol, TEn, folate, methionine, calcium, vitamin D intake
Giovannucci, 1992 [33]	7284 Ca: 170	only M	40–75 (range)	n.a.	colorectal adenoma	n.a.	≥28.3 g/d	RR: 0.36 (0.22–0.60) *p* < 0.0001	Age, TEn, and family history of CRC
Haile, 1997 [34]	Ca: 488 Co: 488	Ca: M = 325 Co: M = 325	Ca: 61.9 ± 6.7 Co: 61.8 ± 6.8	Ca: 19.1 g/d Co: 20.1 g/d	colorectal adenoma	n.a.	27.6 g/d	OR: 0.52 (0.33–0.80) *p* < 0.01	BMI, TEn, PA, smoking and ethnicity
Haslam, 2017 [35]	24,251 Ca: 4063	Ca: M = 1418	55–74 (range)	n.a.	colorectal adenoma	Ca: 796	26.8 ± 10.8 g/d	OR 0.57 (0.52–0.63) *p* < 0.0001	none
Hoff, 1986 [36] (1)	Ca: 23 Co: 77	Ca: M: 16 Co: M = 40	50–59 (range)	Ca: 18.3 ± 1.2 M Co: 21.2 ± 0.9 M	rectal adenoma	n.a.	n.a.	OR: 0.36 (0.12–1.04) *p* = 0.06	none
Hoff, 1986 [36] (2)		Ca: F: 7 Co: F = 37		Ca: 18.9 ± 2.0 F Co: 21.9 ± 0.9 F				OR: 0.34 (0.08–1.51) *p* = 0.16	
Kunzmann, 2015 [37] (1)	19,258 Ca: 1004	Ca: M = 665 Co: M = 8,756	Ca: 66.6 ± 5.0 Co: 66.7 ± 4.9	Ca: 11.2 ± 3.5 g/1000 kcal Co: 12.0 ± 3.6 g/1000 kcal	colorectal adenoma	Ca: 270	≥12.8 g/1000 kcal	OR: 0.76 (0.63–0.91) *p* < 0.003	Age, sex, study center, ethnicity, TEn, smoking status, alcohol intake, total folate intake from diet
Kunzmann, 2015 [37] (2)	Ca: 770				colon adenoma	Ca: 206		OR: 0.75 (0.61–0.92) *p* < 0.006	
Kunzmann, 2015 [37] (3)	Ca: 262				rectal adenoma	Ca: 69		OR 0.68 (0.48–0.96) *p* = 0.03	
Kunzmann, 2015 (a) [37] (4)	Ca: 738 Co: 929	Ca: M = 535 Co: M = 570	Ca: 66.9 ± 5.3 Co: 68.2 ± 5.4	Ca: 11.1 ± 3.4 g/1000 kcal Co: 11.2 ± 3.5 g/1000 kcal	recurrent colorectal adenoma	Ca: 203	≥12.8 g/1000 kcal	OR: 1.08 (0.75–1.55) *p* = 0.67	Age, sex, study center, ethnicity, TEn, smoking status, alcohol intake, total folate intake from diet
Kunzmann, 2015 (a) [37] (5)	Ca: 257				recurrent colon adenoma	Ca: 70		OR: 0.99 (0.59–1.66) *p* = 0.96	
Kunzmann, 2015 (a) [37] (6)	Ca: 78				recurrent rectal adenoma	Ca: 26		OR: 0.88 (0.39–1.99) *p* = 0.86	
Little, 1993 [38]	Ca: 147 Co: 153	Ca: M = 65 Co: M = 65	Ca: 66.0 ± 7.0 Co: 66.0 ± 7.0	Ca: 25.5 g/d Co: 25 g/d	colorectal adenoma	Ca: 26	38 g/d	RR: 0.81 (0.37–1.78) *p* = n.a.	Age, sex, social class and total energy intake
Lubin, 1997 [39]	Ca: 196 Co: 196	Ca: M = 111 Co: M = 111	21–75 (range)	n.a.	colorectal adenoma	n.a.	>34 g/d	OR 0.6 (0.3–1.2) *p* = 0.14	TEn, PA, weight smoking
Martìnez, 1996 [40]	Ca: 157 Co: 480	Ca: M = 98 Co: M = 229	Ca: 57.7 Co: 54.7	n.a.	colorectal adenoma	Ca: 29 Co: 120	28.0–86.8 g/d	OR 0.5 (0.3–0.9) *p* = 0.01	Age, sex, race, BMI, smoking status, family history of CRC, NSAID and aspirin, calcium and fat intake
Mathew, 2004 [41]	Ca: 239 Co: 228	Ca: M = Co: M =	18–74 (range)	n.a.	colorectal adenoma	n.a.	Median (10th–90th percentiles) 6 (4, 11) of energy from various fibres	OR 0.67 (0.45–0.99) *p* = n.a.	Age, sex and TEn
Mujtaba, 2018 [42]	Ca: 789 Co: 2035	Ca: M = 482 Co: M = 871	Ca:58.1 ± 9.2 Co:54.5 ± 10.9	Ca: 21.7 ± 9.4 g/d Co: 22.0 ± 10.1 g/d	colorectal adenoma	Ca: 187	n.a.	OR 1.25 (0.89–1.75) *p* = 0.28	Age, sex, family history of CRC, smoking, alcohol, BMI, height, PA, hormone therapy, aspirin use, NSAID calcium, folate, TEn, total fat, SFA, red and processed meat intake
Nimptsch, 2014 [43]	17,221 Ca: 1299	only F	34–51 (range)	E: 22.0 ± 5.5 g/d nE: 16.7 ± 4.8 g/d	colorectal adenoma	Ca E: 231 Ca nE: 293	n.a.	OR 0.77 (0.65–0.92) *p* = n.a.	TEn
Peters, 2003 [44] (1)	Ca: 3591 Co: 33,971	Ca: M = n.a. Co: M = 17,435	55–74 (range)	21.9 g/d	colorectal adenoma	Ca: 637	30.6 g/d	OR 0.73 (0.62–0.86) *p* < 0.002	Age, sex, study center, TEn
Peters, 2003 [44] (2)	Ca: 2378 Co: 33,971				colon adenoma	Ca: 412		OR 0.70 (0.58–0.85) *p* < 0.0006	
Peters, 2003 [44] (3)	Ca: 659 Co: 33,971				rectal adenoma	Ca: 123		OR 0.93 (0.65–1.33) *p* = 0.97	
Platz, 1997 [45] (1)	16,448 Ca: 690	only M	59.5 ± 9.4	n.a.	colorectal adenoma	Ca: 120 Co: 3171	32.3 g/d	RR = 0.88 (0.72–1.08) *p* = 0.218	Age, endoscopy prior 1986, family history of CRC, BMI, smoking, multivitamin use, PA, aspirin use, alcohol, red meat, folate and methionine
Platz, 1997 [45] (2)	Ca: 531				colon adenoma	Ca: 91		RR: 0.88 (0.59–1.31) *p* = 0.10	
Platz, 1997 [45] (3)	Ca: 159				rectal adenoma	Ca: 29		RR: 1.12 (0.54–2.35) *p* = 0.78	
Sandler, 1993 [46] (1)	Ca: 105 Co: 165	only M	Ca:63.5 ± 12.2 Co:58.7 ± 12.1	n.a.	colorectal adenoma	Ca: n.a.	≥18.6 g/d	OR 0.74 (0.32–1.74) *p* = 0.873	Age, alcohol intake, BMI, and TEn
Sandler, 1993 [46] (2)	Ca: 131 Co: 244	only F	Ca:62.2 ± 11.6 Co:58.7 ± 12.5			Ca: n.a.	≥15.6 g/d	OR 0.71 (0.35–1.43) *p* = 0.120	
Shaw, 2017 [47]	Ca: 1098 no Ca: 1450	Ca: M = 710 no Ca: 675	50–75 (range)	Ca: 10.5–33.59 g/d no Ca: 11.01–33.88 g/d	colorectal adenoma	Ca:205 no Ca: 347	>25.52 g/d	OR 0.77 (0.56–1.07) *p* = 0.14	Age, sex, BMI, smoking, reason for colonoscopy, family history of polyps, TEn
Tantamango, 2011 [48]	2818 Ca: 441	Ca: M = 211 Ca: M = 922	Ca: 73.4 ± 9.2 Co: 71.2 ± 9.7	Ca: 11.2 ± 4.3 g/d Co: 11.7 ± 4.8 g/d	colon adenoma	Ca: 93	16.9 g/d	OR 0.71 (0.51–0.99) *p* = 0.04	Age, sex, BMI, education, PA, alcohol and meat intake
Witte, 1996 [49]	Ca: 488 Co: 488	Ca: M = 334 Co: M = 334	Ca: 61.9 ± 6.7 Co: 61.8 ± 6.8	Ca: 18.9 ± 9.6 g/d Co: 20.0 ± 9.7 g/d	colorectal adenoma	n.a.	n.a.	OR 0.82 (0.65–1.03) *p* = 0.09	None

n.a.: not available; F: Female; M: male; ^ The total sample and number of cases are reported for the cohort study, both the number of Cases (Ca) and Controls (Co) are reported for the case-control study, while the number of Ca and non-cases (no Ca) are reported for the cross-sectional study. BMI: Body Mass Index; Ca/Co: case/control; CRC: Colorectal cancer; IBD: Inflammatory bowel disease; NSAID: Nonsteroidal anti-inflammatory drug; PA: Physical Activity; SAF: Saturated fatty acids; TEn: Total Energy intake.

**Table 3:** Results of the sensitivity and subgroup analyses.

Analysis	Model	*n*. Studies Included	ES	95% CI	*p*	Sample Size	I^2^	*p*	Intercept	Tau (t)	*p*
Trim and Fill colorectal	Fixed *	24	0.71	0.68–0.75	0.000	157,725	62.71	0.000	0.12	0.21	0.838
	Random ^		0.74	0.67–0.82	0.000						
Excluding potential overlapping cohort	Fixed	19	0.79	0.74–0.85	0.000	87,629	13.00	0.295	−0.89	−1.93	0.070
	Random		0.79	0.73–0.86	0.000						
Excluding studies with estimated OR	Fixed	18	0.76	0.71–0.82	0.000	115,311	39.33	0.045	−0.84	−1.41	0.179
	Random		0.74	0.67–0.83	0.000						
9y FU	Fixed	4	0.79	0.71–0.88	0.000	56,453	0.00	0.595	0.15	0.07	0.950
	Random		0.79	0.71–0.88	0.000						
Validated FFQ	Fixed	15	0.71	0.68–0.75	0.000	115,192	74.88	0.000	1.16	1.14	0.276
	Random		0.75	0.66–0.86	0.000						
Diagnosis	Fixed	21	0.70	0.66–0.73	0.000	120,530	64.52	0.000	0.09	0.14	0.891
	Random		0.71	0.63–0.80	0.000						
Quality score ≥ 7	Fixed	20	0.78	0.73–0.83	0.000	125,561	38.10	0.044	−0.99	−1.96	0.065
	Random		0.77	0.70–0.84	0.000						
Colon adenoma	Fixed	4	0.73	0.65–0.83	0.000	74,714	0.00	0.774	1.20	1.20	0.352
	Random		0.73	0.65–0.83	0.000						
Rectal adenoma	Fixed	5	0.77	0.62–0.96	0.019	69,905	30.54	0.218	−0.98	−0.86	0.455
	Random		0.76	0.56–1.03	0.074						
Cohort studies (incidence)	Fixed	10	0.67	0.63–0.71	0.000	135,506	75.38	0.000	1.32	1.12	0.297
	Random		0.72	0.61–0.84	0.000						
Case-Control/Cross-sectional (prevalence)	Fixed	17	0.78	0.72–0.84	0.000	55,401	25.13	0.165	−0.86	−189	0.079
	Random		0.76	0.69–0.85	0.000						
Women	Fixed	6	0.81	0.70–0.92	0.002	35,152	0.00	0.745	−0.50	−1.06	0.349
	Random		0.81	0.70–0.92	0.002						
Men	Fixed	6	0.69	0.58–0.82	0.000	24,426	72.73	0.003	−2.24	−2.72	0.053
	Random		0.46	0.27–0.78	0.004						

* Trimmed studies: 0; ^ Trimmed studies: 2.

## Supplementary Materials

The following are available online at https://www.mdpi.com/article/10.3390/ijerph18084168/s1, Table S1: Full search strategy; Table S2: Studies excluded with reasons after full-text assessment; Table S3: Quality assessment of the included studies.

## References

[1] Wild C.P., Weiderpass E., Stewart B.W., International Agency for Research on Cancer (2020). World Cancer Report: Cancer Research for Cancer Prevention.

[2] Schreuders E.H., Ruco A., Rabeneck L., Schoen R.E., Sung J.J., Young G.P., Kuipers E.J. (2015). Colorectal cancer screening: A global overview of existing programmes. Gut.

[3] Centres for Diseases Control and Prevention Colorectal Cancer Screening Tests. https://www.cdc.gov/cancer/colorectal/basic_info/screening/tests.htm.

[4] Fleming M., Ravula S., Tatishchev S.F., Wang H.L. (2012). Colorectal carcinoma: Pathologic aspects. J. Gastrointest Oncol..

[5] World Cancer Research Fund Diet, Nutrition, Physical Activity and Colorectal Cancer. https://www.wcrf.org/sites/default/files/Colorectal-cancer-report.pdf.

[6] Gianfredi V., Salvatori T., Villarini M., Moretti M., Nucci D., Realdon S. (2018). Is dietary fibre truly protective against colon cancer? A systematic review and meta-analysis. Int. J. Food Sci. Nutr..

[7] Gianfredi V., Nucci D., Salvatori T., Dallagiacoma G., Fatigoni C., Moretti M., Realdon S. (2019). Rectal Cancer: 20% Risk Reduction Thanks to Dietary Fibre Intake. Systematic Review and Meta-Analysis. Nutrients.

[8] Higgins J.P., Altman D.G., Gotzsche P.C., Juni P., Moher D., Oxman A.D., Savovic J., Schulz K.F., Weeks L., Sterne J.A. (2011). The Cochrane Collaboration’s tool for assessing risk of bias in randomised trials. BMJ.

[9] Stroup D.F., Berlin J.A., Morton S.C., Olkin I., Williamson G.D., Rennie D., Moher D., Becker B.J., Sipe T.A., Thacker S.B. (2000). Meta-analysis of observational studies in epidemiology: A proposal for reporting. Meta-analysis Of Observational Studies in Epidemiology (MOOSE) group. JAMA.

[10] Moher D., Liberati A., Tetzlaff J., Altman D.G., Group P. (2009). Preferred reporting items for systematic reviews and meta-analyses: The PRISMA statement. PLoS Med..

[11] Liberati A., Altman D.G., Tetzlaff J., Mulrow C., Gotzsche P.C., Ioannidis J.P., Clarke M., Devereaux P.J., Kleijnen J., Moher D. (2009). The PRISMA statement for reporting systematic reviews and meta-analyses of studies that evaluate health care interventions: Explanation and elaboration. Ann. Intern. Med..

[12] Higgins J.P.T., Green S. (2013). Cochrane Handbook for Systematic Reviews of Interventions. Version 5.1.0..

[13] Brown P., Brunnhuber K., Chalkidou K., Chalmers I., Clarke M., Fenton M., Forbes C., Glanville J., Hicks N.J., Moody J. (2006). How to formulate research recommendations. BMJ Clin. Res. Ed..

[14] Gianfredi V., Salvatori T., Nucci D., Villarini M., Moretti M. (2018). Can chocolate consumption reduce cardio-cerebrovascular risk? A systematic review and meta-analysis. Nutrition.

[15] Gianfredi V., Bragazzi N.L., Nucci D., Villarini M., Moretti M. (2017). Cardiovascular diseases and hard drinking waters: Implications from a systematic review with meta-analysis of case-control studies. J. Water Health.

[16] Wells G.A., Shea B., O’Connell D., Paterson J., Welch V., Losos M., Tugwell P. The Newcastle-Ottawa Scale (NOS) for Assessing the Quality of Nonrandomised Studies in Meta-Analyses. http://www.ohri.ca/programs/clinical_epidemiology/oxford.asp.

[17] Herzog R., Alvarez-Pasquin M.J., Diaz C., Del Barrio J.L., Estrada J.M., Gil A. (2013). Are healthcare workers’ intentions to vaccinate related to their knowledge, beliefs and attitudes? A systematic review. BMC Public Health.

[18] Egger M., Davey Smith G., Schneider M., Minder C. (1997). Bias in meta-analysis detected by a simple, graphical test. BMJ Clin. Res. Ed..

[19] Duval S., Tweedie R. (2000). A nonparametric ”Trim and Fill” method of accounting for Publication Bias in Meta-Analysis. J. Am. Stat. Assoc..

[20] Leimu R., Koricheva J. (2004). Cumulative meta-analysis: A new tool for detection of temporal trends and publication bias in ecology. Proc. Biol. Sci..

[21] Austin G.L., Adair L.S., Galanko J.A., Martin C.F., Satia J.A., Sandler R.S. (2007). A diet high in fruits and low in meats reduces the risk of colorectal adenomas. J. Nutr..

[22] Benito E., Cabeza E., Moreno V., Obrador A., Bosch F.X. (1993). Diet and colorectal adenomas: A case-control study in Majorca. Int. J. Cancer.

[23] Haslam A., Wagner Robb S., Hébert J.R., Huang H., Ebell M.H. (2018). Association between dietary pattern scores and the prevalence of colorectal adenoma considering population subgroups. Nutr. Diet. J. Dietit. Assoc. Aust..

[24] Kopp T.I., Vogel U., Tjonneland A., Andersen V. (2018). Meat and fiber intake and interaction with pattern recognition receptors (TLR1, TLR2, TLR4, and TLR10) in relation to colorectal cancer in a Danish prospective, case-cohort study. Am. J. Clin. Nutr..

[25] Kune G.A., Kune S., Read A., MacGowan K., Penfold C., Watson L.F. (1991). Colorectal polyps, diet, alcohol, and family history of colorectal cancer: A case-control study. Nutr. Cancer.

[26] Macquart-Moulin G., Riboli E., Cornee J., Kaaks R., Berthezene P. (1987). Colorectal polyps and diet: A case-control study in Marseilles. Int. J. Cancer.

[27] Neugut A.I., Garbowski G.C., Lee W.C., Murray T., Nieves J.W., Forde K.A., Treat M.R., Waye J.D., Fenoglio-Preiser C. (1993). Dietary risk factors for the incidence and recurrence of colorectal adenomatous polyps. A case-control study. Ann. Intern. Med..

[28] Zheng X., Nguyen L.H., Liu P.H., Wu K., Smith-Warner S., Willett W., Chan A.T., Giovannucci E., Cao Y. (2019). Comprehensive assessment of diet quality and risk of early-onset colorectal adenoma. Gastroenterology.

[29] Breuer-Katschinski B., Nemes K., Marr A., Rump B., Leiendecker B., Breuer N., Goebell H., Colorectal Adenoma Study G. (2001). Colorectal adenomas and diet: A case-control study. Colorectal Adenoma Study Group. Dig. Dis. Sci..

[30] Byrd D.A., Judd S., Flanders W.D., Hartman T.J., Fedirko V., Bostick R.M. (2020). Associations of Novel Dietary and Lifestyle Inflammation Scores with Incident, Sporadic Colorectal Adenoma. Cancer Epidemiol. Biomarkers Prev..

[31] Fu Z., Shrubsole M.J., Smalley W.E., Ness R.M., Zheng W. (2014). Associations between dietary fiber and colorectal polyp risk differ by polyp type and smoking status. J. Nutr..

[32] Fuchs C.S., Giovannucci E.L., Colditz G.A., Hunter D.J., Stampfer M.J., Rosner B., Speizer F.E., Willett W.C. (1999). Dietary fiber and the risk of colorectal cancer and adenoma in women. N. Engl. J. Med..

[33] Giovannucci E., Stampfer M.J., Colditz G., Rimm E.B., Willett W.C. (1992). Relationship of diet to risk of colorectal adenoma in men. J. Natl. Cancer Inst..

[34] Haile R.W., Witte J.S., Longnecker M.P., Probst-Hensch N., Chen M.J., Harper J., Frankl H.D., Lee E.R. (1997). A sigmoidoscopy-based case-control study of polyps: Macronutrients, fiber and meat consumption. Int. J. Cancer.

[35] Haslam A., Wagner Robb S., Hebert J.R., Huang H., Wirth M.D., Shivappa N., Ebell M.H. (2017). The association between Dietary Inflammatory Index scores and the prevalence of colorectal adenoma. Public Health Nutr..

[36] Hoff G., Moen I.E., Trygg K., Frolich W., Sauar J., Vatn M., Gjone E., Larsen S. (1986). Epidemiology of polyps in the rectum and sigmoid colon. Evaluation of nutritional factors. Scand. J. Gastroenterol..

[37] Kunzmann A.T., Coleman H.G., Huang W.Y., Kitahara C.M., Cantwell M.M., Berndt S.I. (2015). Dietary fiber intake and risk of colorectal cancer and incident and recurrent adenoma in the Prostate, Lung, Colorectal, and Ovarian Cancer Screening Trial. Am. J. Clin. Nutr..

[38] Little J., Logan R.F., Hawtin P.G., Hardcastle J.D., Turner I.D. (1993). Colorectal adenomas and diet: A case-control study of subjects participating in the Nottingham faecal occult blood screening programme. Br. J. Cancer.

[39] Lubin F., Rozen P., Arieli B., Farbstein M., Knaani Y., Bat L., Farbstein H. (1997). Nutritional and lifestyle habits and water-fiber interaction in colorectal adenoma etiology. Am. Assoc. Cancer Res..

[40] Martinez M.E., McPherson R.S., Annegers J.F., Levin B. (1996). Association of diet and colorectal adenomatous polyps: Dietary fiber, calcium, and total fat. Epidemiology.

[41] Mathew A., Peters U., Chatterjee N., Kulldorff M., Sinha R. (2004). Fat, fiber, fruits, vegetables, and risk of colorectal adenomas. Int. J. Cancer.

[42] Mujtaba S., Bostick R.M. (2018). Differences in risk factor-colorectal adenoma associations according to non-steroidal anti-inflammatory drug use. Eur. J. Gastroenterol. Hepatol..

[43] Nimptsch K., Malik V.S., Fung T.T., Pischon T., Hu F.B., Willett W.C., Fuchs C.S., Ogino S., Chan A.T., Giovannucci E. (2014). Dietary patterns during high school and risk of colorectal adenoma in a cohort of middle-aged women. Int. J. Cancer.

[44] Peters U., Sinha R., Chatterjee N., Subar A.F., Ziegler R.G., Kulldorff M., Bresalier R., Weissfeld J.L., Flood A., Schatzkin A. (2003). Dietary fibre and colorectal adenoma in a colorectal cancer early detection programme. Lancet.

[45] Platz E.A., Giovannucci E., Rimm E.B., Rockett H.R., Stampfer M.J., Colditz G.A., Willett W.C. (1997). Dietary fiber and distal colorectal adenoma in men. Cancer Epidemiol. Biomarkers Prev..

[46] Sandler R.S., Lyles C.M., Peipins L.A., McAuliffe C.A., Woosley J.T., Kupper L.L. (1993). Diet and risk of colorectal adenomas: Macronutrients, cholesterol, and fiber. J. Natl. Cancer Inst..

[47] Shaw E., Warkentin M.T., McGregor S.E., Town S., Hilsden R.J., Brenner D.R. (2017). Intake of dietary fibre and lifetime non-steroidal anti-inflammatory drug (NSAID) use and the incidence of colorectal polyps in a population screened for colorectal cancer. J. Epidemiol. Community Health.

[48] Tantamango Y.M., Knutsen S.F., Beeson L., Fraser G., Sabate J. (2011). Association between dietary fiber and incident cases of colon polyps: The adventist health study. Gastrointest. Cancer Res..

[49] Witte J.S., Longnecker M.P., Bird C.L., Lee E.R., Frankl H.D., Haile R.W. (1996). Relation of vegetable, fruit, and grain consumption to colorectal adenomatous polyps. Am. J. Epidemiol..

[50] U.S. Department of Agriculture, U.S. Department of Health and Human Services (2020). Dietary Guidelines for Americans, 2020–2025.

[51] Angelino D., Godos J., Ghelfi F., Tieri M., Titta L., Lafranconi A., Marventano S., Alonzo E., Gambera A., Sciacca S. (2019). Fruit and vegetable consumption and health outcomes: An umbrella review of observational studies. Int. J. Food Sci. Nutr..

[52] Gianfredi V., Vannini S., Moretti M., Villarini M., Bragazzi N.L., Izzotti A., Nucci D. (2017). Sulforaphane and Epigallocatechin Gallate Restore Estrogen Receptor Expression by Modulating Epigenetic Events in the Breast Cancer Cell Line MDA-MB-231: A Systematic Review and Meta-Analysis. J. Nutr..

[53] Gianfredi V., Nucci D., Vannini S., Villarini M., Moretti M. (2017). In vitro Biological Effects of Sulforaphane (SFN), Epigallocatechin-3-gallate (EGCG), and Curcumin on Breast Cancer Cells: A Systematic Review of the Literature. Nutr. Cancer.

[54] EFSA Panel on Dietetic Products Nutrition and Allergies (NDA) (2010). Scientific Opinion on Dietary Reference Values for carbohydrates and dietary fibre. EFSA J..

[55] Macfarlane G.T., Macfarlane S. (2012). Bacteria, colonic fermentation, and gastrointestinal health. J. AOAC Int..

[56] Canani R.B., Costanzo M.D., Leone L., Pedata M., Meli R., Calignano A. (2011). Potential beneficial effects of butyrate in intestinal and extraintestinal diseases. World J. Gastroenterol..

[57] Iebba V., Totino V., Gagliardi A., Santangelo F., Cacciotti F., Trancassini M., Mancini C., Cicerone C., Corazziari E., Pantanella F. (2016). Eubiosis and dysbiosis: The two sides of the microbiota. New Microbiol..

[58] Lobionda S., Sittipo P., Kwon H.Y., Lee Y.K. (2019). The Role of Gut Microbiota in Intestinal Inflammation with Respect to Diet and Extrinsic Stressors. Microorganisms.

[59] Ahn J., Sinha R., Pei Z., Dominianni C., Wu J., Shi J., Goedert J.J., Hayes R.B., Yang L. (2013). Human gut microbiome and risk for colorectal cancer. J. Natl. Cancer Inst..

[60] FAO (Food and Agriculture Organization), WHO (World Health Organization) (1998). Carbohydrates in Human Nutrition.

[61] Williams B.A., Mikkelsen D., Flanagan B.M., Gidley M.J. (2019). “Dietary fibre”: Moving beyond the “soluble/insoluble” classification for monogastric nutrition, with an emphasis on humans and pigs. J. Anim. Sci. Biotechnol..

[62] World Cancer Research Fund, American Institute for Cancer Research (2018). Diet., Nutrition, Physical Activity and Cancer: A Global Perspective.

[63] Bach-Faig A., Berry E.M., Lairon D., Reguant J., Trichopoulou A., Dernini S., Medina F.X., Battino M., Belahsen R., Miranda G. (2011). Mediterranean diet pyramid today. Science and cultural updates. Public Health Nutr..

[64] Fiolet T., Srour B., Sellem L., Kesse-Guyot E., Alles B., Mejean C., Deschasaux M., Fassier P., Latino-Martel P., Beslay M. (2018). Consumption of ultra-processed foods and cancer risk: Results from NutriNet-Sante prospective cohort. BMJ Clin. Res. Ed..

[65] Monteiro C.A., Cannon G., Moubarac J.C., Levy R.B., Louzada M.L.C., Jaime P.C. (2018). The UN Decade of Nutrition, the NOVA food classification and the trouble with ultra-processing. Public Health Nutr..

[66] Agus A., Denizot J., Thevenot J., Martinez-Medina M., Massier S., Sauvanet P., Bernalier-Donadille A., Denis S., Hofman P., Bonnet R. (2016). Western diet induces a shift in microbiota composition enhancing susceptibility to Adherent-Invasive *E. coli* infection and intestinal inflammation. Sci. Rep..

[67] Garcia-Montero C., Fraile-Martinez O., Gomez-Lahoz A.M., Pekarek L., Castellanos A.J., Noguerales-Fraguas F., Coca S., Guijarro L.G., Garcia-Honduvilla N., Asunsolo A. (2021). Nutritional Components in Western Diet Versus Mediterranean Diet at the Gut Microbiota-Immune System Interplay. Implications for Health and Disease. Nutrients.

[68] Yu G., Bei J., Zhao J., Li Q., Cheng C. (2018). Modification of carrot (Daucus carota Linn. var. Sativa Hoffm.) pomace insoluble dietary fiber with complex enzyme method, ultrafine comminution, and high hydrostatic pressure. Food Chem..

[69] Wang Y., Sun P., Li H., Adhikari B.P., Li D. (2018). Rheological Behavior of Tomato Fiber Suspensions Produced by High Shear and High Pressure Homogenization and Their Application in Tomato Products. Int. J. Anal. Chem..

