# Supplementary material for: Association between Dietary Fibre Intake and Colorectal Adenoma: A Systematic Review and Meta-Analysis
Source: Int J Environ Res Public Health. 2021 Apr 15;18(8):4168. doi: 10.3390/ijerph18084168 (PMC8071151; doi:10.3390/ijerph18084168)
Supplement: Supplementary file 1 [file ijerph-18-04168-s001.zip › ijerph-1150683-supplementary.pdf]

Supplementary

Table S1. Full search strategy.

SET	PubMed/MEDLINE
1	“Dietary Fiber“ [Mesh:NoExp]
2	“Bread“ [Mesh]
3	“Edible Grain“ [Mesh]
4	“Fruit“ [Mesh]
5	“Vegetables“ [Mesh]
6	Fiber [tiab]
7	Fibre [tiab]
8	Sets 1-7 were combined with “OR”
9	“Colorectal Neoplasms“ [Mesh]
10	“Colonic Neoplasms“ [Mesh]
11	“Rectal Neoplasms“ [Mesh]
12	“Intestinal Polyps“ [Mesh]
13	“Adenomatous Polyps“ [Mesh]
14	Sets 9-13 were combined with “OR”
15	“Prospective Studies“ [MeSH Terms]
16	“Cross-Sectional Studies“ [MeSH Terms]
17	“Follow-Up Studies“ [MeSH Terms]
18	“Surveys and Questionnaires“ [MeSH Terms]
19	“Incidence“ [MeSH Terms]
20	“Cohort Studies"[MeSH Terms]
21	“epidemiology”[MeSH Terms]
22	Sets 15-21 were combined with “OR”
23	Sets 8, 14 and 22 were combined with “AND”

Table S2. Studies excluded with reasons after full-text assessment.

Author Year [Ref]	Number	Reasons for Exclusion
Austin, 2007; Kune, 1991; Haslam A, 2018; Kopp 2018; Zheng, 2019	5	No data specifically referred to dietary fibre intake
Benito, 1993; Macquat, 1987; Neugut, 1993	3	Data non-extractable

Table S3. Quality assessment of the included studies.

**Author year [Ref]**	**Selection**				**Comparability**		**Outcome/Exposure^**			**Total**
	Item 1	Item 2	Item 3	Item 4	Item 5a	Item 5b	Item 6	Item 7	Item 8	**score/9**
**Breuer-Katschinski, 2001**	*	*	-	*	-	*	-	*	*	6
Breuer-Katschinski, 2001 (a)	*	*	*	*	-	*	-	*	*	7
Byrd, 2020	*	*	*	*	-	-	*	*	*	7
Fu, 2014	*	*	*	*	*	*	*	*	*	9
Fuchs, 1999	-	*	*	*	*	*	*	*	-	7
Giovannucci, 1992	*	*	*	*	*	*	*	-	*	8
Haile, 1997	*	*	*	*	-	*	*	*	*	8
Hoff, 1986	*	*	*	*	-	-	-	*	-	5
Haslam, 2017	*	*	*	*	-	-	*	-	-	5
Kunzmann, 2015	*	*	*	*	*	*	*	*	*	9
Little, 1993	*	*	*	*	*	*	-	*	-	7
Lubin, 1997	*	*	*	*	-	*	*	*	*	8
Martinez, 1996	*	*	*	*	*	*	*	*	-	8
Mathew, 2004	*	*	*	*	*	*	*	*	*	9
Mujtaba, 2018	*	*	*	*	*	*	*	*	*	9
Nimptach, 2014	-	*	*	*	-	*	*	*	-	6
Peters, 2003	*	*	*	*	*	*	*	*	-	8
Platz, 1997	*	*	*	*	*	*	*	*	-	8
Sandler, 1993	*	*	*	*	-	*	*	*	-	7
Shaw, 2017	*	*	*	*	*	*	*	*	-	8
Tantamago, 2011	-	*	-	*	*	*	-	*	-	5
Witte, 1996	*	*	*	*	-	-	*	*	*	7

^ Based on study design. The NOS assessed exposure for case-control and the outcome for cross-sectional and cohort studies.
